# MiR-211 is essential for adult cone photoreceptor maintenance and visual function

**DOI:** 10.1038/s41598-017-17331-z

**Published:** 2017-12-05

**Authors:** Sara Barbato, Elena Marrocco, Daniela Intartaglia, Mariateresa Pizzo, Sabrina Asteriti, Federica Naso, Danila Falanga, Rajeshwari S. Bhat, Nicola Meola, Annamaria Carissimo, Marianthi Karali, Haydn M. Prosser, Lorenzo Cangiano, Enrico Maria Surace, Sandro Banfi, Ivan Conte

**Affiliations:** 1 Telethon Institute of Genetics and Medicine, Via Campi Flegrei 34, Pozzuoli (Naples), 80078 Italy; 2 Department of Translational Research, University of Pisa, Via San Zeno 31, 56123 Pisa, Italy; 3 Medical Genetics, Department of Biochemistry, Biophysics and General Pathology, University “Luigi Vanvitelli”, via Luigi De Crecchio 7, 80138 Naples, Italy; 4 The Wellcome Trust Sanger Institute, Hinxton, Cambridge, CB10 1SA United Kingdom; 5 Department of Translational Medicine, University of Naples Federico II, Naples, Italy; 6 Present Address: Aarhus University, Department of Molecular Biology and Genetics, C.F. Møllers Allé 3 building 1130, 422–8000 Aarhus C, Denmark; 7 Present Address: Department of Physiology, Development and Neuroscience, University of Cambridge, Cambridge, CB2 3EG United Kingdom

## Abstract

MicroRNAs (miRNAs) are key post-transcriptional regulators of gene expression that play an important role in the control of fundamental biological processes in both physiological and pathological conditions. Their function in retinal cells is just beginning to be elucidated, and a few have been found to play a role in photoreceptor maintenance and function. MiR-211 is one of the most abundant miRNAs in the developing and adult eye. However, its role in controlling vertebrate visual system development, maintenance and function so far remain incompletely unexplored. Here, by targeted inactivation in a mouse model, we identify a critical role of miR-211 in cone photoreceptor function and survival. MiR-211 knockout (−/−) mice exhibited a progressive cone dystrophy accompanied by significant alterations in visual function. Transcriptome analysis of the retina from *miR-211*−/− mice during cone degeneration revealed significant alteration of pathways related to cell metabolism. Collectively, this study highlights for the first time the impact of miR-211 function in the retina and significantly contributes to unravelling the role of specific miRNAs in cone photoreceptor function and survival.

## Introduction

The vertebrate retina is a layered structure composed of six neuronal types and glial cells, including Müller cells, astrocytes and microglia. Among the different neuronal cell types, photoreceptors are the primary sensory neurons that carry out phototransduction and initiate vision. They include two different types, namely rods and cones, with the rods being responsible for dim light (i.e., night) vision and the cones being necessary for bright light (i.e. daylight) colour vision with high temporal and spatial acuity. Cone photoreceptors constitute about 3–5% of photoreceptor cells in rodents and humans. In humans, cone function may be specifically and primarily impaired by mutations in some genes (most of which display cone-restricted expression) leading to diseases, termed cone dystrophies, characterized by significant impairment of central vision and eventually blindness. Cone dystrophies have an estimated prevalence of 1:30,000–40,000 and may be broken down into two broad groups - stationary and progressive^1^. However, cone dysfunction and/or loss can also be secondary to rod degeneration. Retinitis Pigmentosa (RP), diabetic retinopathy and Age-related Macular Degeneration (AMD) are the most common disorders characterized by secondary cone alterations in humans^1–5^. The specific cellular mechanisms underlying cone death are poorly understood, although several hypotheses, not necessarily mutually exclusive, have been raised. It has been proposed that secondary cone cell death may derive from lack of survival or neurotrophic factors, nutrient deprivation due to an abnormal contact with rods, retinal pigment epithelium (RPE), or Müller glia^6,7^. Alternatively, release of toxic metabolites by dying rods, oxidative stress due to retinal hyperoxia, and “collateral damage” caused by activated microglia may also play a role^1,6,7^. More recently, proof-of-concept studies are shedding new light on the pathological mechanisms behind secondary cone cell death suggesting a main role for the rod-derived cone viability factor (RdCVF), a survival factor protecting cones from secondary degeneration^8^. Overall a full understanding of the genetic causes and of the molecular mechanisms underlying primary and secondary cone death still represents a challenge but nevertheless is a prerequisite in the development of strategies aimed at preserving vision in retinal degenerative diseases.

MicroRNAs (miRNAs) are a class of 20- to 25-nucleotide small noncoding RNA molecules that have basic roles in post-transcriptional regulation of gene expression and represent key players in the control of fundamental biological processes in both physiological and pathological conditions^9^. Evidence indicates that miRNAs are important for the development and maintenance of correct function within the eye, in particular the retina^10–13^. More specifically, recent findings have shown that several miRNAs are required to prevent loss of adult cone photoreceptor outer segments and visual function^13,14^. Notably, inactivation of the miR-182/183/96 cluster results in syndromic photoreceptor degeneration^15^. However, the consequences of the lack of single miRNAs with respect to photoreceptor maintenance and survival are still unknown.

Among the miRNAs abundantly expressed in the visual system, the two members of the miR-204/211 family show an evolutionarily conserved expression in many eye districts, particularly in the retinal pigment epithelium (RPE), the neural retina, the lens, and the ciliary body^16,17^. So far, the most extensively studied member of this family is miR-204^10,16–22^. We recently found that miR-204 plays a crucial role in the differentiation and function of all of the ocular structures in which it is expressed^10,16–19^. This miRNA is involved in the correct differentiation and function of RPE cells and is also required for proper lens development^16,17^. Many gene networks modulated by miR-204 have been discovered, including the Meis2-mediated control of the transcription factor Pax6, a “master regulator” of eye development^16,17^. Notably, we also identified a point mutation in miR-204 with a causative role in inherited retinal dystrophies in human patients, which represents not only the first retinal disease caused by mutations in miRNAs but also provides the first example of a microRNA-caused genetic disease likely occurring via a gain-of-function mechanism^10^.

Despite the pervasive role of miR-204 in many aspects of eye development and function, to date, there is very limited information on the function in the eye of its closely related paralog miR-211. In most mammals, including mouse, miR-211 only shows one nucleotide difference with the mature sequence of miR-204. The two miRNAs share an identical seed region, i.e., a sequence element of approximately 7 nucleotides which is reported to play a key role in mRNA target recognition^23^. This suggests that both miRNAs target a similar set of genes. Preliminary data suggest that *miR-211* expression is controlled by the transcription factor MITF and that this miRNA could contribute to RPE differentiation^24^. Here, by analysing the functional consequences of miR-211 loss-of-function in mice, we show that this miRNA is essential for cone photoreceptor maintenance and function. This is the first study to reveal the direct involvement of miR-211 in the visual system, extending the functional role of this miRNA family to cone photoreceptors.

## Results

### Characterization of *miR-211*−/− mice

*MiR-211* is one of the most abundant miRNAs in the retina^25^ and its expression is sensitive to light levels^26^. High resolution expression data in the eye for this miRNA have been previously reported only in the adult retina^26^. By RNA *in situ* hybridization (ISH), we showed that the expression of *miR-211* is abundant in the developing eye and is detected in the presumptive RPE, ciliary body, distal region of the developing retina of the optic cup thus recapitulating the expression of its host gene, the melastatin, transient receptor potential cation channel subfamily M member 1 (*Trpm1*) gene (Supplementary Fig. 1). In the adult eye, *miR-211* is expressed in the RPE and in the Inner Nuclear Layer (INL) with a weaker expression detected in the Outer Nuclear Layer (ONL) and in the Ganglion Cell Layer (GCL) as previously reported^26^ (Supplementary Fig. 2c,d), again recapitulating the expression pattern of its host gene^27^.

To explore miR-211 function *in vivo*, we generated a miR-211-deficient mouse line in which the precursor sequence of *miR-211* (*pre-miR-211*) was deleted by homologous recombination^28^ (Supplementary Fig. 2a). *MiR-211*−/− mice were viable and appeared to be normal. We performed quantitative RT-PCR (qPCR) assay for *miR-211* using total RNA from the retina of postnatal day (P)30 wild-type and *miR-211*−/− mice. We found that the mature *miR-211* was not detectable in the retinas of all *miR-211*−/− mice that we examined (n = 3) (Supplementary Fig. 2b). Concordantly, by RNA ISH we detected no *miR-211* expression in the retina of *miR-211*−/− mice at P30 (Supplementary Fig. 2c–e). However, its paralog miR-204 was expressed (Supplementary Fig. 2f). As previously stated, *miR-211* is an intragenic miRNA, which is located in an intronic region, i.e., between exon 6 and exon 7, of the *Trpm1* gene^29^. Therefore, we asked whether the deletion of pre-miR-211 could have any effect on the proper expression of *Trpm1*, which could contribute to the phenotype of *miR-211*−/− mice. Western blot analysis showed no altered expression of any of the *Trpm1* isoforms upon *miR-211* deletion in the retina (Supplementary Fig. 3a,b). In retina protein extracts, a major band of 180 kDa was detectable together with a band of 110 kDa, a band of 70 kDa and one of 55 kDa^30^. These observations together with the qRT-PCR analysis (Supplementary Fig. 3c) demonstrated that in *miR-211*−/− eyes, the expression levels of *Trpm1* was not altered when compared with WT animals. Moreover, we detected no alteration in *Trpm1* mRNA maturation and splicing by both RT-PCR (Supplementary Fig. 3d) and qRT-PCR analyses (Supplementary Fig. 3e) further confirming the specificity of miR-211 depletion.

### *MiR-211*−/− mice display a progressive cone dystrophy

**Figure 1:**
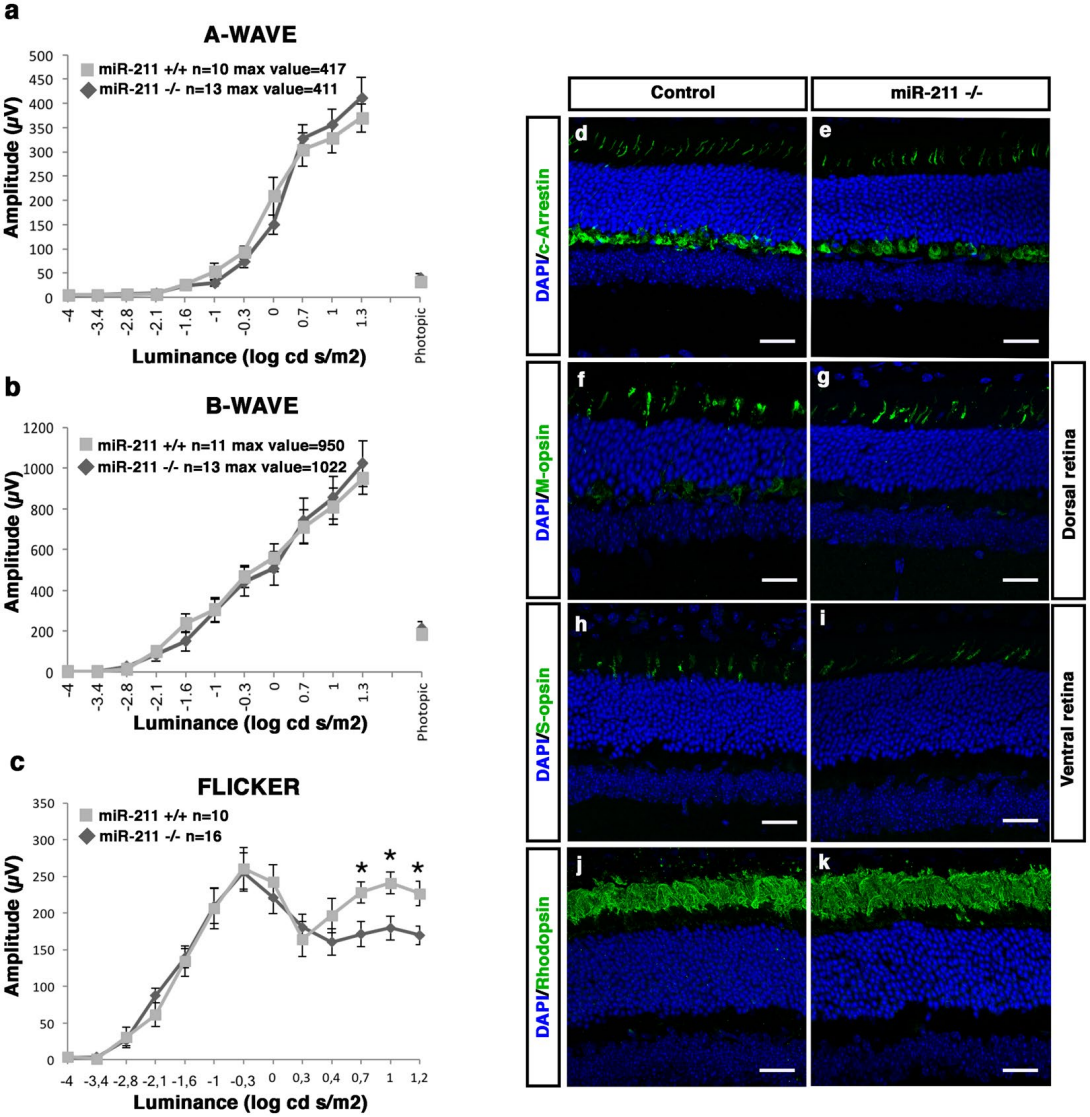
One month old *miR-211*−/− mice display a normal expression pattern of photoreceptor markers but initial signs of cone dysfunction. **(a**,**b)** ERG responses, plotted as a function of stimulus intensity, from one month wild type (grey squares) and *miR-211*−/− (black rhombi) mice. The amplitudes of the dark-adapted, scotopic a-wave and b-wave of the *miR-211*−/− mice are similar to the amplitudes of the wt control mice. **(c)** Flicker responses, plotted as a function of stimulus intensity, from one month old wt (grey squares) and *miR-211*−/− (black rhombi) mice. Scotopic indicates flashes of 20.0 cd s/m^2^ light intensity; photopic indicates flashes of 20.0 cd s/m^2^ light intensity on a constant background illumination of 50 cd/m^2^; Flicker recordings were performed with light intensities ranging from 10^−4^ to 15 cd s/m^2^ in steps of 0.6 logarithmic units at 6 Hz frequency. Error bars represent SEM. *P Value < 0.05 t test. **(d**–**k)** Representative immunofluorescence images of control **(d**,**f**,**h**,**j)** and *miR-211*−/− **(e,g,i,k)** retinas at one month, stained with different retinal markers (green) and DAPI (blue). No differences are visible in the sections stained with the photoreceptor markers cone-Arrestin **(d,e)**, M-opsin **(f,g)**, S-opsin **(h,i)** and Rhodopsin **(j,k)**, Scale bars: 50 μm.

**Figure 2:**
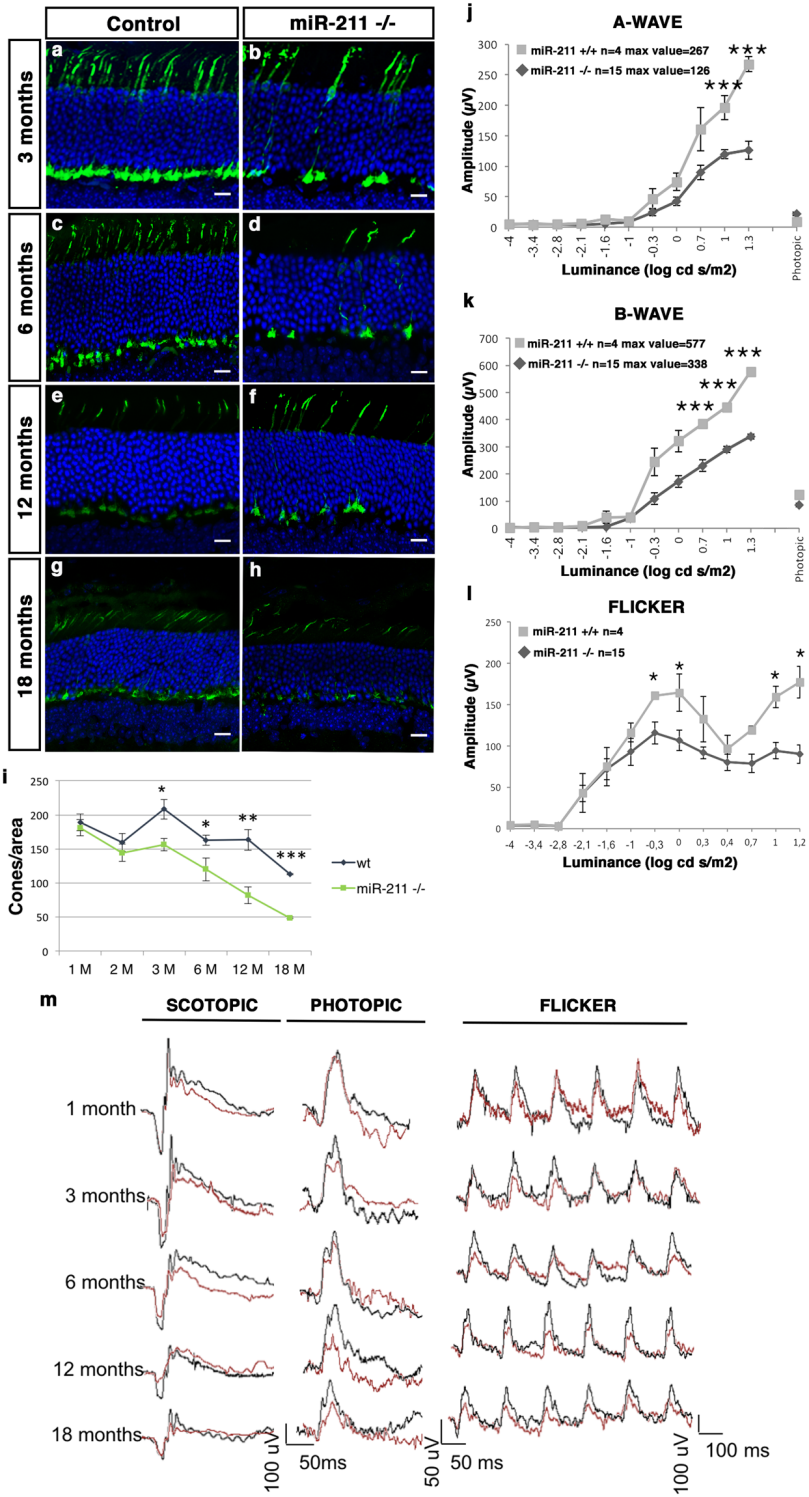
miR-211 deletion results in progressive morphological and functional defects in cones. (**a**–**h**) Representative immunofluorescence images of control **(a,c,e,g)** and *miR-211*−/− **(b,d,f,h)** retinas at three months **(a,b)**, six months **(c,d)**, twelve months **(e,f)** and eighteen months (**g,h**) stained with cone-Arrestin antibody (green) and DAPI (blue). Scale bars: 50 μm. **(i**) Graphs showing cone density (cones/area). From three months of age onward, there are significant differences in the number of cones between wt and *miR-211*−/− mice up to a greater than 50% reduction in aged *miR-211*−/− mice. Error bars represent SEM. ***P Value < 1 × 10^−5^, **P Value < 0.01, *P Value < 0.05 t test. (**j**–**k**) ERG responses, plotted as a function of stimulus intensity, from wt (grey squares) and *miR-211*−/− (black rhombi) mice, at eighteen months of age. The amplitudes of the dark-adapted, scotopic a-wave and b-wave of *miR-211*−/− mice are significantly lower at higher intensities than in wt control mice. Error bars represent SEM. ***P Value < 0.001, **P Value < 0.01 t test. **(l**) Flicker responses, plotted as a function of stimulus intensity, from wt (grey squares) and *miR-211*−/− (black rhombi) mice, at eighteen months of age. The b-wave amplitude of *miR-211*−/− mice is significantly lower than in wt mice in the light intensity ranges that stimulate only cone and rod-cone responses. Error bars represent SEM. *P Value < 0.05, **P Value < 0.01 t test. **(m**) Representative ERG (scotopic and photopic responses) and flicker traces at 1, 3, 6, 12 and 18 months of age show the progressive decrease of ERG and flicker responses of *miR-211*−/− mice (red lines) compared to wt controls mice (black lines; Fig. 1 and Supplementary Fig. 6). Scotopic indicates flashes of 20.0 cd s/m^2^ light intensity; photopic indicates flashes of 20.0 cd s/m^2^ light intensity on a constant background illumination of 50 cd/m^2^; Flicker recordings were performed with light intensities ranging from 10^−4^ to 15 cd s/m^2^ in steps of 0.6 logarithmic units at 6 Hz frequency.

To explore the phenotypic consequences of *miR-211* deletion, we carried out a detailed analysis of *miR-211* homozygous mutant mice. Therefore, we performed a morphological analysis at two postnatal stages: undifferentiated retina at P1 and the fully differentiated retina at P30. We did not identify any apparent morphological alteration in any ocular structures at P1 (Supplementary Fig. 2g–j). At P30, we first evaluated retinal function by standard electroretinographic (ERG) recordings in *miR-211*−/− mice. We found that both photopic and scotopic responses in *miR-211*−/− mice were unchanged compared to WT mice (Fig. 1a,b). In addition, to further investigate rod and cone responses, we took advantage of the 6-Hz scotopic flicker ERG paradigm^31^. This protocol enables the isolation of rod from cone responses by varying light intensities (ranging from −5 to 2 log cd s/m^2^), while maintaining 6 Hz stimulation frequency^31^. *MiR-211*−/− mice showed considerable reduction of the amplitudes at light intensities typically dominated by cone responses (Fig. 1c, highest light intensities). Thus, we then examined whether cones and rods degenerate in *miR-211*−/− mice at P30. This analysis was carried out by quantifying cone and rod density on retinal sections that were labelled by immunostaining with anti-cone Arrestin, anti-S-opsin, anti-M-opsin and anti-Rhodopsin antibodies, which mark cones, blue cones, green cones and rods, respectively. We did not detect any significant decrease in cone and rod densities in *miR-211−/−*, compared to WT control mice (Figs 1d–k and 2i, Supplementary Fig. 4f,g).

Overall, the above data suggest the presence of signs of cone dysfunction in 1-month old *miR-211*−/− mice in spite of apparently normal photoreceptor morphology. We then extended our morphological and functional analysis to later ages, focusing on 3-, 6-, 12- and 18-month-old animals (Fig. 2, Supplementary Fig. 4, Supplementary Fig. 5 and Supplementary Fig. 6). In *miR-211*−/− mice, we observed a progressive decrease in the amplitudes at highest light intensities, compared to WT control mice (Fig. 2j,k,m and Supplementary Fig. 6a,b,d,e,g,h). Considering that light intensities beyond −2.0 log cd s/m2 evoke responses originated by both rod and cone photoreceptors (mesopic range)^32^, these data support the possibility that the observed ERG decrease may affect mainly the cone-derived components. Indeed, previous work on* Rho−/−* mice, in which rods are non-functioning, shows pure cone responses in the mesopic range (and unpublished ERG recording, E.M.S. and E.M. data)^32,33^. Notably, the cone photoreceptor responses, as assessed by 6-Hz scotopic flicker analysis, showed a progressive reduction from 3-month to 18-month-old *miR-211*−/− mice reaching approximately a 50% amplitude reduction in 18-month old mice as compared to age-matched wild-type controls (Fig. 2l,m and Supplementary Fig. 6c,f,i). This progression was also observed in cone ERG photopic responses (Supplementary Fig. 6b,e,h). However, a slight difference at low light intensity in the serial flicker recordings was also observed suggesting that rod involvement may not be excluded in the absence of further electrophysiological evaluation such as rod-bipolar patch clamp analysis.

Consistent with a progressive and ultimately severe functional cone deficiency observed from the age of 3-months onward, *miR-211*−/− mice showed a gradual reduction of cone density at these same stages (Fig. 2i and Supplementary Fig. 4a–e). Notably, 18-month old *miR-211*−/− mice showed approximately a 50% decrease of cone density compared to age-matched wild type controls (Fig. 2g–i). Crucially, these changes were not associated with defects in rod density or a significant reduction in ONL thickness (Supplementary Fig. 4f,g), further supporting the observation that miR-211 depletion induces a cone-specific dystrophy. We also performed the TUNEL assay to detect cone cell death in the retina of *miR-211*−/− mice at 3-, 6-, and 12-months of age. Notably, we did not observe any significant increase in TUNEL-positive cells in *miR-211*−/−, compared with WT mice (Supplementary Fig. 7a–l). The latter result may be explained by both the slow rate of cone degeneration observed in *miR-211*−/− mice and by the low abundance of cones, which represent the 3% of photoreceptor cells in the retina that can preclude the detection of cone cell death. Overall, these data indicate that miR-211 deletion in mice leads to a progressive cone dystrophy phenotype.

### *MiR-211* ablation leads to a specific cone dysfunction

**Figure 3:**
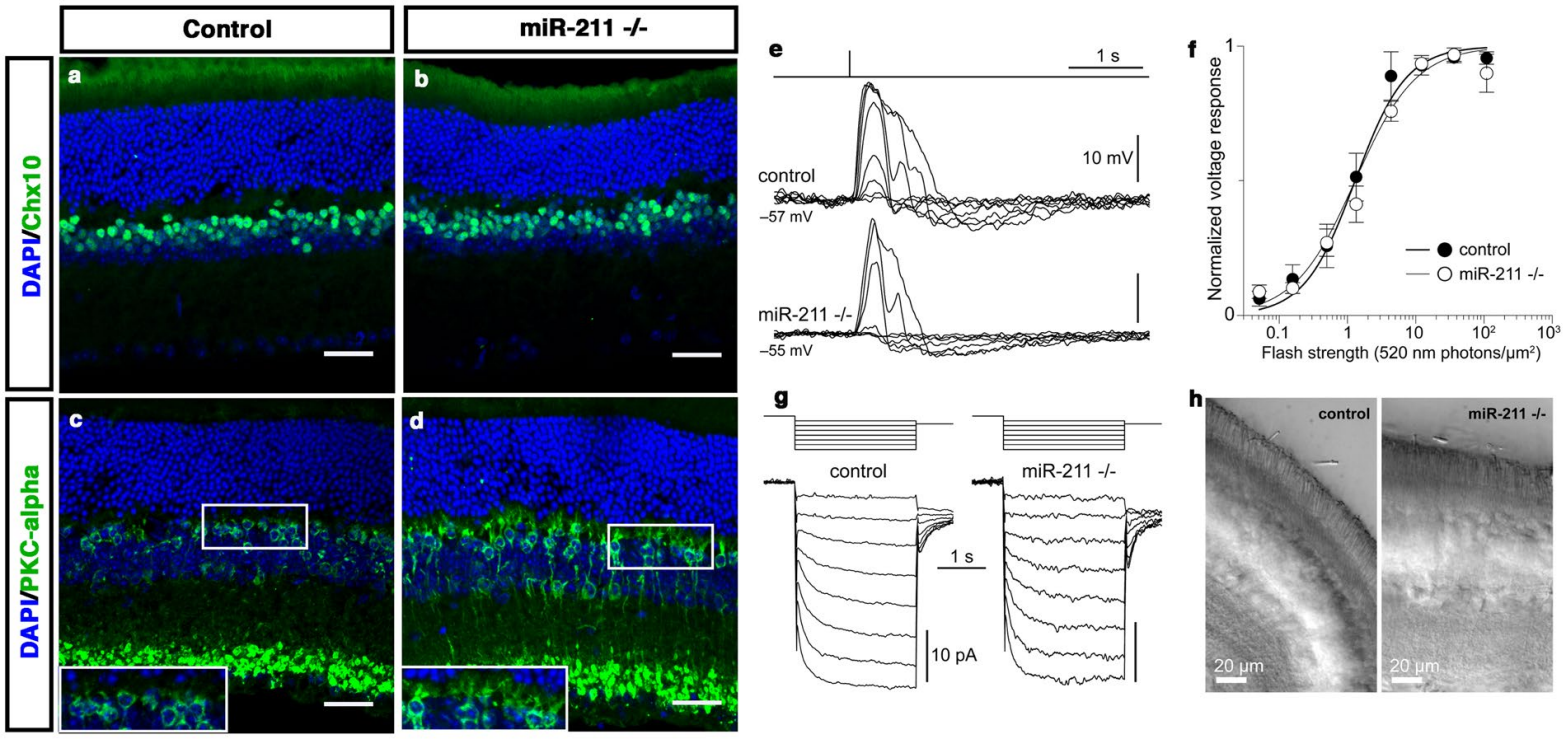
MiR-211 deletion does not affect rod and rod bipolar cell function. Representative immunofluorescence images of wild type (**a,c**) and *miR-211*−/− (**b,d**) mouse retinas at 7 months, stained with anti-Chx10 (**a,b**) and anti-PKC-alpha (**c,d**) antibodies. In *miR-211*−/− retinas the pattern of expression of the bipolar cell markers analysed is similar to wt mice. However, a slight remodelling of aberrant processes of the PKC-α-bipolar positive cells was observed to extend through the ONL of the retina of miR-211−/− (magnified in white boxes). Scale bars: 50 μm. **(e)** Representative current-clamp responses to 520 nm flashes of increasing strength (0.051, 0.16, 0.50, 1.3, 4.3, 12, 36, 109 photons/µm^2^) from a control and a *miR-211*−/− rod bipolar cell (RBC). Dark membrane potentials are shown beside the records (uncorrected for junction or Donnan potentials). **(f)** Summary plot of the normalized flash response amplitudes vs. flash strength from control (black circles; n = 6) and *miR-211*−/− RBCs (white circles; n = 6). Means, SEMs and Hill function fits are shown. Half saturating flash strengths, a measure of light sensitivity, do not differ significantly between the two groups (p = 0.11; extra-sum-of-squares F-test). **(g)** Representative voltage-clamp responses to hyperpolarizing voltage steps (−53 mV to −60/−67/−74/−81/−88/−95/−102/−109 mV to −65 mV) from a control and a *miR-211*−/− RBC. The records show clear hyperpolarization-activated cyclic nucleotide-gated currents (I_h_), normally present in RBCs. **(h)** Live retinal slices from control and *miR-211*−/− mice, visualized with infrared microscopy during recording, show an intact appearance of rod outer segments (cone outer segments are too few and too small to be resolved). All recordings were made in dark adapted retinal slices from 7-month old animals (see Methods for details).

Two observations suggest that miR-211 could have additional functions in other retinal cell types, apart from cone photoreceptors. First, RNA ISH expression data indicate that *miR-211* is highly expressed in the RPE, INL and in the GCL (Supplementary Fig. 2c). Second, *miR-211* seems to be co-expressed with the *Trpm1* gene, which is known to play a relevant role in bipolar cell functionality^34–36^. In that respect, mutations in the human *TRPM1* gene lead to defects in the depolarizing light response of ON-bipolar cells^37–39^ and cause congenital stationary night blindness in patients^37–39^. These observations prompted us to evaluate the possible consequences of miR-211 ablation in bipolar cells as well as in other retina cell types. Towards this goal, we first carried out immunofluorescence analysis to assess retinal cell morphology. Analysis of the expression of the Chx10 (bipolar), PKC-α (rod bipolar), GS (Müller), Pax6 (amacrine and ganglion cells) and Calbindin (horizontal) markers, did not reveal any significant defects in other adult retinal cell types in *miR-211*−/− mice (7 months old) when compared with age-matched controls (Fig. 3a–d and Supplementary Fig. 8). However, a slight remodelling of aberrant processes of the PKC-α-bipolar positive cells was observed to extend through the ONL of the retina of *miR-211−/−* (Fig. 3), as a consequence of cone dystrophy^40,41^. As previously mentioned, the alterations of the mesopic response detected by ERG analysis in *miR-211*−/− mice (Figs 1 and 2, Supplementary Fig. 6) do not rule out some rod involvement. To exclude such a possibility, we recorded the scotopic response of depolarizing (ON) rod bipolar cells (RBCs) of 7-month old mice with the perforated patch clamp technique. Since 20–25 rods converge on each RBC, this neuron would be strongly impacted by a dysfunction in rods. Moreover, recording from the RBCs ensured that we avoided any measurement-induced perturbations of the small and delicate rod photoreceptors^42^. Remarkably, the RBCs from *miR-211*−/− mice did not show any significant alteration from the behaviour of age matched controls in terms of light sensitivity, photovoltage amplitude and ion channel expression (Fig. 3e–h). These results further confirmed the specificity of the cone dystrophy phenotype. Some forms of cone dystrophies can be the consequence of impaired retinal circulation^43^. Interestingly, previous studies have shown that alteration of *miR-211* expression was associated with increase of vascular invasion in cancer^44^. Therefore, we asked whether the retinal vasculature was impaired in *miR-211*−/− mice. Fundus examinations by fluorangiography and *in vivo* fluorescein isothiocyanate (FITC)-dextran assays revealed no abnormalities in the superficial, connecting, or deep vascular layers of retinal circulation of the *miR-211*−/− mice compared with control mice (Supplementary Fig. 9). Moreover, we also asked whether the RPE morphology was impaired. Notably, comparison of the morphology of the differentiated RPE/retina of control and *miR-211*−/− mice did not reveal significant alteration in thickness and laminar organization (Supplementary Fig. 9k–l). Altogether, these data suggest a relevant and specific role of miR-211 in the control of cone photoreceptor survival and function.

### MiR-211 coordinates expression of genes relevant to retinal metabolism

**Figure 4:**
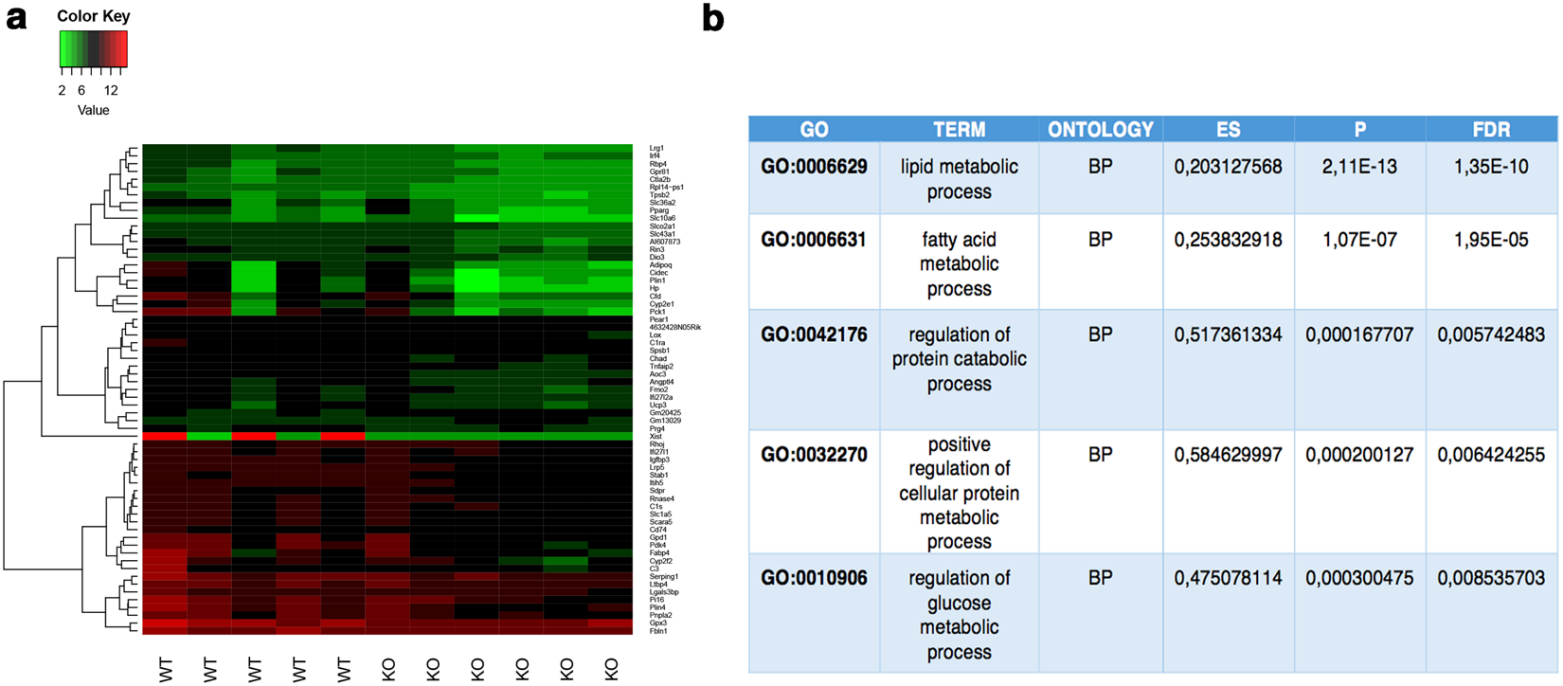
Differential expression analysis and enriched GO categories of miRNA targets. **(a)** Heatmap showing gene expression profiles of the differentially expressed genes in *miR-211*−/− vs. wild type retinas (FDR < 0.1). Each column represents an independent replicate. Expression values are row normalized and color–coded (red indicates maximal value, green indicates minimal value for each gene). **(b)** Selection of Gene Ontology terms significantly enriched by using GSEA. The table displays the Enrichment Score (ES), p-value and False Discovery Rate (FDR) derived from the analysis of each gene set.

To gain insight into the molecular basis underlying the miR-211-induced phenotype, we decided to carry out transcriptome analysis of *miR-211*−/− mice by RNAseq. To increase the likelihood of detecting the transcriptome changes related to the functional phenotype and not those secondary to cone cell death, RNA-Seq libraries were prepared from whole eye, after removal of the lens and cornea from 1–2 month old *miR-211*−/− mice, stages in which cone cell loss was not yet detected. RNA-Seq libraries were prepared for each eye sample in multiple biological replicates, five for WT and six for *miR-211*−/− animals. Each library was sequenced using 100 bp paired-end sequencing on the Illumina HiSeq. 1000 system. Gene abundances from RNA-Seq data were quantified using RSEM^45^. This approach yielded read count values for a total of 38253 mouse genes annotated in GenCode^46^. We only considered genes that had at least 1 count per million in at least five out of 11 samples as expressed, yielding a total of 15590 genes. Next we performed differential gene expression analysis to determine the transcriptional effects of miR-211 deletion. This analysis yielded a total of 63 genes that were differentially expressed with a False Discovery Rate (FDR) < 0.1 (Fig. 4). Of these, the expression levels of 61 genes were significantly decreased upon miR-211 deletion, while only 2 genes were upregulated (listed in Supplementary Table 1). qRT-PCR analysis on 4 of top differentially expressed genes (Supplementary Fig. 7) showed a concordant trend with RNA-seq results without reaching statistical significance. We then performed Gene Set Enrichment Analysis (GSEA)^47^ for the GO gene-sets derived from Biological Process, Cellular Component and Molecular Function Ontology as annotated in the MsigDB Database^47^. We obtained an Enrichment Score (ES) and an adjusted p-value for each gene-set. We observed a highly significant (FDR < 0.05) enrichment for gene-sets related to metabolic and catabolic processes (Fig. 4). Moreover, a gene ontology enrichment analysis using DAVID^48^ revealed that an important proportion of both downregulated genes were related to cellular metabolism and catabolism, including pyruvate and glucose (i.e., *Rbp4*, *Gpd1*, *Pdk4*, *Adipoq*, *Pck1*), protein and lipid metabolic processes (i.e., *C1ra*, *C3, Cfd* and *Ucp3, Fabp4, Adipoq*, respectively). Interestingly, alterations of glucose metabolism have already been shown in a non-cell autonomous fashion, to impact on cone function^13,49^. Therefore, our data suggest that the ablation of miR-211 leads to a metabolic status change of retinal cells that may contribute to the cone dystrophy observed in *miR-211*−/− mice.

## Discussion

A considerable amount of information has been recently gathered on the key roles of miRNAs in the regulation of fundamental cellular processes, such as cell fate determination, cell death and proliferation, and also on the recognition of the miRNAs displaying the most abundant (and sometimes cell layer-restricted) levels of expression in either the developing or the adult retina. Conditional inactivation of miRNA biogenesis, through knockout of the *Dicer1* and *Dgcr8* genes revealed that miRNAs are necessary for proper photoreceptor development and function in mice^11,14,50–52^. In particular, the ablation of *Dicer1* highlighted multiple activities of miRNAs in the maintenance of post mitotic rod photoreceptor (PR) cells^11^. Moreover, cone-specific disruption of *Dgcr8* in mice has been associated with the loss of adult cone outer segments, resulting in reduction of light responses^14^. Nevertheless, there is only limited information on the roles of individual miRNAs on photoreceptor differentiation, maintenance and function. In particular, re-expression of miR-182 and miR-183 in cone conditional *Dgcr8*−/− mice prevented outer segment loss suggesting their requirement for cone outer segment maintenance^14^. Moreover, targeted deletion in mice of the *retinal non-coding RNA 3* (*Rncr3*) gene, the predominant source of miR-124, resulted in specific apoptosis of newly differentiated cone photoreceptors, which was partially rescued after re-expression of miR-124^13^.

One of the miRNAs for which at present there are more data concerning the direct involvement in photoreceptor cell specification, differentiation and survival is miR-204. Recently, we reported a pathogenic mutation within miR-204 as the cause of an ocular disease characterised by coloboma associated with retinal dystrophy^10^. We also demonstrated that ablation of miR-204 function in medaka fish leads to progressive alteration and death of photoreceptor cells, suggesting a direct function of miR-204 in differentiation, maintenance and function of these cells^10^. In mammals, miR-204 has a closely related paralog, miR-211 by which it only differs by one or two nucleotides in the mature sequence, depending on the species^17^. The two miRNAs share the same seed-region sequence and therefore are predicted to target a similar set of genes (TargetScan^53^, although, in the latter respect, precise information is still lacking). *MiR-211* first appeared in mammals through the evolution of one of the two copies of miR-204, which is present in two identical copies in the genomes of early vertebrates and fish, including Medaka fish^16,17^. Interestingly, we found that *miR-211* is expressed in the RPE, INL, GCL and at lower levels in photoreceptor cells in the adult retina, recapitulating in full the expression pattern of its host gene previously reported in the human retina^27^. To date, however, miR-211 function in the eye has not been thoroughly analysed and its *in vivo* role remains unclear.

Here, we establish, for the first time, the existence of a specific relationship between miR-211 activity and cone function and survival in mammals. In particular, we found that miR-211 inactivation in mouse leads to a progressive cone dysfunction followed by cone loss, without the apparent involvement of rods. Among the evidence supporting the latter conclusion, a special emphasis should be placed on the lack of a decrease in ONL thickness, even in very old mice, and the unaltered light sensitivity of RBCs assessed with patch clamp at 7-months. The extent of cone loss is of about 50% at late ages (18 months). It is possible that the residual cone survival and function might be explained by a partial functional redundancy of the paralog miR-204. The latter miRNA, since it shares with miR-211 the same seed region, and therefore is predicted to target a similar set of genes, can in principle compensate at least in part the lack of miR-211. However, recent findings also highlighted a distinct and context-dependent targeting activities exerted by the miR-204 and miR-211 paralogs^54^. The cone loss observed in *miR-211*−/− mice could be a consequence of increased cell death, as also suggested by previous studies that have shown that miR-211 can directly regulate these events by modulating *Chop* expression^55^. However, we did not find any alteration in *Chop* expression or in the related pathway (Supplementary Fig. 7m). We also were not able to detect any TUNEL-positive cells in the retina of *miR-211*−/− mice (Supplementary Fig. 7a–l), reflecting a very slow degeneration of cones, which are not highly abundant in the mouse retina.

Interestingly, our transcriptome analysis suggests that miR-211 could be involved in controlling the metabolism and catabolism of retinal cells, including genes participating in glucose, pyruvate and lipid metabolic process. The possibility of a miR-211-mediated control of retinal metabolism is particularly attractive; indeed, previous reports have proposed that the paralog miR-204 is able to block insulin production during the diabetic process^56^, in which a progressive retinopathy is frequently associated as secondary pathological hallmark. In the adult retina, the Rod-derived Cone Viability Factor (RdCVF), an inactive thioredoxin secreted factor, is considered to be a survival factor as it is primarily required for the stimulation of glucose metabolism, preventing secondary cone death in models of RP^8^. Interestingly, the thioredoxin-interacting protein (TXNIP) was reported to regulate the miR-204-MAFA-insulin pathway contributing to glucose metabolism and diabetes progression^56^. However, miR-211 activity might be MAFA-independent, because no alteration of the MafA mRNA was observed in the *miR-211*−/− transcriptome. Moreover, we also detect additional metabolic pathways such as pyruvate and lipid metabolic processes, suggesting that miR-211 might function as a metabolic switch factor controlling the homeostasis of miR-211-expressing cells. Indeed, an abnormal regulation of energy metabolism has been recently reported for miR-211 in human melanoma cells^57^. Notably, alterations of both pyruvate and lipid metabolisms are strictly associated with oxidative stress and retinal dystrophy^58–62^.

Although the effects of miR-211 inactivation seem to be mostly restricted to cone photoreceptor cells, yet this miRNA does not seem to display high levels of expression in this cell type. However, we hypothesize, based also on the above reported considerations, that this apparent conundrum may be explained by the high susceptibility of cones to alterations of retinal metabolism/catabolism, as also previously reported. Interestingly, the paralogous *miR-204* has also been previously demonstrated to play a key role in photoreceptor function and maintenance in spite of its low expression in photoreceptor cells (REF). All these observations further support the evidence that miRNAs can exert important roles even in cell types in which they display relatively low expression levels^10,63,64^. However, we cannot exclude that cone dysfunction in *miR-211*−/− mice might at least in part be also due to non-cell autonomous effects. Overall, the mode of action of miR-211 and its relevance in the control of retinal metabolism and catabolism processes as well as in the function of other retinal cell types (e.g., bipolar cells in which miR-211 is particularly abundant) might be more complex and require further study.

In summary, we have begun to dissect the function of miR-211 in eye development and function by demonstrating that it plays an important role in cone photoreceptor maintenance and function. Given the importance of the miR-204/211 miRNA family in photoreceptor function and disease, it will be of the utmost relevance to identify the targets of both miRNAs to unravel their important roles in visual physiology and pathogenesis.

## Materials and Methods

### Generation of miR-211 knockout mice

The *miR-211* knockout mouse line (mmu-mir-211−/−) employed in this study was generated by the Wellcome Trust Sanger Institute^28^. The strain was obtained by deleting 160 bp (containing the pre-miR-211) from the wild type sequence. All studies on animals were conducted in strict accordance with the institutional guidelines for animal research and approved by the Italian Ministry of Health; Department of Public Health, Animal Health, Nutrition and Food Safety in accordance to the law on animal experimentation (article 7; D.L. 116/92; protocol number: 00001/11/IGB; approval date June 6, 2011). Furthermore, all animal treatments were reviewed and approved in advance by the Ethics Committee of Ospedale Cardarelli (Naples, Italy). *MiR-211*−/− mice were maintained on the C57Bl/6J background. In all experiments, we used as controls aged-matched littermates of *miR-211*−/− mice.

### Quantitative Real Time PCR

For wild type and *miR-211*−/− mice analysis, the RNAs were extracted from whole eyes. All the RNAs were extracted and digested with DNaseI using RNeasy extraction kit (Qiagen) according to the manufacturer’s instructions. The cDNAs were generated using the QuantiTect Reverse Transcription Kit (Qiagen) for the qRT-PCR analysis. The qRT-PCR reactions were performed with nested primers and carried out with the Roche Light Cycler 480 system. The PCR reaction was performed using cDNA (200–500 ng), 10 µl of the SYBR Green Master Mix (ROCHE) and 400 nM primers, in a total volume of 20 µl. The PCR conditions for all the genes were as follows: preheating, 95 °C for 5 min; cycling, 40 cycles of 95 °C for 15 s, 60 °C for 15 s and 72 °C for 25 s. Quantified results were expressed in terms of cycle threshold (Ct). The Ct values were averaged for each triplicate. The murine *Hprt* gene was used as the endogenous control for the experiments. Differences between the mean Ct values of the tested genes and those of the reference gene were calculated as DCtgene = Ctgene - Ctreference. Relative expression was analysed as 2^−DCt^. Relative fold changes in expression levels were determined as 2^−DDCt^ ^65^. The sequences of oligonucleotide primers are summarized in Supplementary Table 2.

### Immunofluorescence and ISH on sections

Immunofluorescence analysis was performed on 12 µM cryosections as described previously^17^, using the following primary antibodies: mouse anti-Rhodopsin (1:5000, Abcam ab3267), rabbit anti-Cone-Arrestin (1:1000, EMD Millipore AB15282), rabbit anti-M-Opsin (1:200, Santa Cruz sc-30022), rabbit anti-S-Opsin (1:200, Santa Cruz sc-30021), rabbit anti-Chx10 (1:500, Santa Cruz sc-365519), rabbit anti-Pax6 (1:250, Hybridoma Bank 901301), rabbit anti-PCKα (1:250, Sigma P4334), mouse anti-GS6 (1:200, EMD Millipore MAB302), rabbit anti-Calbindin D-28K (1:300, EMD Millipore AB1778). Secondary antibodies were conjugated to Alexa 594 goat anti-rabbit/mouse (1:1000, Invitrogen A-11037, A-11032) or Alexa 488 goat anti-rabbit/mouse (1:1000, Invitrogen A-11008, A-11001). When necessary, slides were permeabilized with boiling in Sodium Citrate Buffer (8,3 ml Sodium Citrate 1 M; 1,9 ml Citric Acid 1 M; water to 200 ml) (α-Chx10; α-Pax6; α-PKCα; α-GS6; α-M-Opsin; α-S-Opsin). For the cone-Arrestin protocol, permeabilization was performed with NP40 1% for 15 minutes at room temperature. For Calbindin staining, retinas were rinsed 3 times in PBS for 30 minutes and blocked for 2 hours at room temperature (RT) in blocking solution [3% goat serum/1% bovine serum albumin (BSA)/0.1% Triton-X100/0.02% sodium dodecyl sulfate (SDS) in PBS]. Retinas were then incubated in primary antibody in blocking solution overnight at 4 °C on a rocking platform. The next day, retinas were rinsed 3 times in PBS for 30 minutes and placed in secondary antibody in blocking solution 3 hours at RT on a rocking platform. Retinas were rinsed 3 times in PBS for 30 minutes and then flattened between two coverslips for confocal imaging. The slides were counterstained with 4,6-diamidino-2-phenylindol, DAPI (Vector Laboratories H-1200). Slides were imaged using the LSM710 Zeiss Confocal Microscopy system. RNA *in situ* hybridization on mouse sections was performed according to previously published protocols^10^. *In situ* hybridization probes for the murine *Trpm1* were generated by reverse transcriptase (RT-) PCR on adult eye cDNA using the oligonucleotide primers listed in Supplementary Table 2. Both mmu-miR-211 miRCURY LNA™ Detection probe, 5′-DIG and 3′-DIG labelled and scramble-miR miRCURY LNA™ Detection probe, 5′-DIG and 3′-DIG labelled as negative control (working concentration 1/150 mM/ml, cod. 616391-360 miR-211 and 699004-360, Exiqon,) were hybridized to frozen sections as described previously^66^. Coverslips were mounted onto glass slides with 70% glycerol in PBS or dehydrated before mounting (Eukitt Mounting Medium; EMS, Fort Washington, PA).

### Western blotting

Proteins were extracted from eyes of wild type and *miR-211*−/− mice by homogenization in RIPA buffer. The extracted proteins (30 µg) were subjected to SDS-PAGE and blotted onto a PVDF membrane (EMD Millipore IPVH00010). After blocking with 1% BSA in TBS, membrane filters were incubated with an anti-TRPM1 antibody (1:1000, Abcam 72154) and anti-ß-Actin antibody (1:700, Sigma A5441). Immuno-signals were visualized with a horseradish peroxidase (HRP)-conjugated anti-rabbit/mouse IgG antibody (1:10000, EMD Millipore 12-348/12-349). Western blot bands were revealed using a chemiluminescence digital imaging system (ImageQuant Las-4000 Mini, GE Healthcare Life Sciences) and quantified using ImageJ software.

### Electrophysiological testing

#### Electroretinogram (ERG)

We used previously described procedures^67^. For electrophysiological recordings (ERGs), mice were dark-adapted for 3 hours and anesthetized. ERGs were evoked by 10 ms flashes of different light intensities ranging from 10^−4^ to 20 cd s/m^2^ generated through a Ganzfeld stimulator (CSO, Florence, Italy). ERGs and b-wave thresholds were assessed using the following protocol: eyes were stimulated with light flashes increasing from −5.2 to +1.3 log cd s/m^2^ (which corresponds to 1 × 10^−5.2^ to 20.0 cd s/m^2^) in scotopic conditions. The log unit interval between stimuli was 0.3 log from −5.4 to 0.0 log cd s/m^2^, and 0.6 log from 0.0 to +1.3 log cd s/m^2^. For ERG analysis in scotopic conditions the responses evoked by 11 stimuli (from −4 to +1.3 log cd s/m^2^) with an interval of 0.6 log unit were considered. To minimize the noise, three ERG responses were averaged at each 0.6 log unit stimulus from −4 to 0.0 log cd s/m^2^ while one ERG response was considered for higher (0.0 – +1.3 log cd s/m^2^) stimuli. The time interval between stimuli was 10 s from −5.4 to 0.7 log cd s/m^2^, 30 s from 0.7 to +1 log cd s/m^2^, or 120 s from +1 to +1.3 log cd s/m^2^. a- and b-wave amplitudes recorded in scotopic conditions were plotted as a function of increasing light intensity (from −4 to +1.3 log cd s/m^2^). The photopic ERG was recorded after the scotopic session by stimulating the eye with 10 flashes of 10 ms with 20.0 cd s/m^2^ light intensity on a constant background illumination of 50 cd/m^2^.

#### Flicker

Flashes of different light intensities ranging from 10^−4^ to 15 cd s/m^2^ in steps of 0.6 logarithmic units at 6 Hz frequency were generated by a Ganzfeld stimulator (CSO, Florence, Italy). Amplitudes of b-wave were plotted as a function of increasing light intensity, at a constant 6 Hz frequency, as already reported in the Seeliger protocol^31^.

#### Patch clamp recordings

Dissection procedures and perforated patch clamp recordings of rod bipolar cells in mouse retinal slices were performed as described^68–70^. Briefly, 7-months old mice were dark adapted for at least 3 hours. Retinas extracted in cold bicarbonate-buffered AMES’ medium (A1420; Sigma-Aldrich, St. Louis, MO, USA) were made to adhere on filter paper with gentle suction and sliced on a manual tissue chopper (mod. 600; The Vibratome Company, St. Louis MO) at a thickness of 250 µm. After transferring the slices to the recording chamber they were superfused with bicarbonate-buffered AMES’ at 24 °C and visualized with an upright microscope (Leica DM-LFSA) in the near infrared spectrum (LED with peak emission at 780 nm). Seals were obtained on rod bipolar cell somata located in the outer third of the inner nuclear layer using 6–9 MΩ patch pipettes, filled with a solution containing (in mM): 90 potassium aspartate, 20 K_2_SO_4_, 15 KCl, 10 NaCl, 5 K_2_Pipes, and 0.4 mg/ml Amphotericin-B; final pH was adjusted to 7.2 with HCl/KOH. Seals were followed immediately by patch perforation and intracellular access. Rod bipolar cells were identified by the shape and position of their soma, light sensitivity and polarity and ion channel expression^68^. Recordings were made with an Axopatch 1D amplifier, low–pass filtered at 500 Hz, digitized at 5 kHz and acquired by pClamp software (Axon Instruments, Foster City CA). Membrane potentials were uncorrected for liquid junction and Donnan potentials. Full field flashes of flashes of 1 to 10 ms duration were delivered with a green LED (OD520; Optodiode Corp., Newbury Park CA) with its emission peak at 520 nm.

### Blood vessel analysis

#### Fluorangiography

Intraperitoneal injection of 1% fluorescein sodic solution (Sigma F7505) was performed in mice after pupil dilatation (1% tropicamide). Fundus photographs were captured by Topcon TRC-50IX (Topcon) in order to display the retinal vasculature as described^71^.

#### FITC-Dextran assay

After anaesthesia, mice were perfused through the left ventricle with 2 ml of PBS containing 1 mg/ml of fluorescein-dextran-FITC (Sigma 60842-46-8). The eyes were then enucleated and fixed in 4% paraformaldehyde in PBS for 2 hours at RT. After RPE removal, flat mount retinas were viewed under a fluorescence microscope and imaged using the LSM710 Zeiss Confocal Microscopy system as described^71^.

### Hematoxylin and Eosin staining

The staining was performed on 7 µm mouse retina paraffin embedded sections. The sections were deparaffinized and rehydrated with 2 × 10′ Xylene; 1 × 5′ 100% ethanol; 1 × 5′ 95% ethanol; 1 × 5′ 90% ethanol; 1 × 5′ 70% ethanol; 1 × 5′ deionized H2O. Hematoxalin staining: 1 × 1′ Hematoxalin; Rinse deionized water; 1 × 5′ Tap water (to allow stain to develop). Eosin staining and dehydration: 1 × 1′ Eosin; 3 × 5′ 70% ethanol; 3 × 5′ 90% ethanol; 3 × 5′ 95% ethanol; 3 × 5′ 100% ethanol; 3 × 10′ Xylene. Slides were coverslipped using Eukitt (Sigma).

### Detection of apoptotic cell death

The extent and distribution of apoptotic cell death was determined by TdT-mediated dUTP nick end labelling (TUNEL), using the *In Situ* Cell Death Detection Kit, POD (Roche 11684817910) following the manufacturer’s directions. TUNEL assay was performed on 20 µm mouse retina cryosections. As negative control to evaluate possible unspecific results, fixed and permeabilized retina sections were incubated with the reaction mix without TUNEL reaction enzyme. As positive control, Apl1 −/− mouse retina cryosections were used. The Apl1 −/− mouse is a model characterized by a high level of photoreceptor cell death^72,73^. Sections were observed with a Leica DM-6000 microscope and then confocal images were acquired using the LSM710 Zeiss Confocal Microscopy system.

### Cone photoreceptor cell counts, assessment of ONL density and thickness

#### Cone cell counts

Two groups of six mice for both *miR-211*−/− and *miR-211*+/+ genotypes, were analysed for each time point. At least twelve retina sections from each eye sample from both genotypes were immunostained with the cone marker cone-Arrestin and counterstained with the DAPI nuclear acid stain. Evaluation of the number of cones was performed by counting the cells positive for cone-Arrestin staining in standard areas of comparable regions in respect to the distance from the optic nerve. The number of cones/area was evaluated by manual counts with a Leica DM-6000 microscope, with the objective Leica ∞/0.17/D, HCX PL FLUOTAR, 40X/0.75 that has an area of 0.31 mm^2^.

#### Retinal cell counts and ONL thickness measurement

The same retina sections on which cones were counted were imaged by confocal microscopy using a Leica TCS SPE 40x objective, using Z-stacks in the wavelength of DAPI. Then the images were used to calculate the number of the ONL nuclei (ONL cell density) and ONL thickness. The evaluation of the number of ONL nuclei was performed by counting the DAPI stained nuclei in areas of comparable regions in respect to the distance from the optic nerve. The selection of each area and the nuclei counts were manually done, using the *ITEM Analysis Image Processing* program that stores and then processes the results. Finally, the ONL thickness was manually measured close to the optic nerve head, in both superior and inferior retina using the *ITEM Analysis Image Processing* program^72,73^. In addition, at least twelve retina sections from each eye sample from both genotypes were used for the evaluation of the number of bipolar, amacrine and ganglion cells. The analysis was performed by counting the cells positive for α-Chx10; α-Pax6 and α-PKCα staining in standard areas of comparable regions in terms of the distance from the optic nerve. The number of retinal cell/area was evaluated by manual counts with a Leica DM-6000 microscope, with the objective Leica ∞/0.17/D, HCX PL FLUOTAR, 40X/0.75 that has an area of 0.31 mm^2^.

Moreover, the number of horizontal cells was evaluated by manual counts of the cells positive for α-Calbindin on at least three flat mount retina from both genotypes.

### RNA extraction, library preparation and sequencing

We used previously described procedures^25^. Total RNA was extracted from the mouse eye including retina, RPE, Choroid, sclera and ciliary body, whereas the lens and cornea were removed. RNA was extracted with the miRNeasy Kit (QIAGEN 217004) according to the manufacturer’s instructions, and quantified using a NanoDrop ND-8000 spectrophotometer (NanoDrop Technologies). Its integrity was evaluated using an RNA 6000 Nano chip on a Bioanalyzer (Agilent Technologies). RNA-Seq libraries were prepared for each RNA sample in biological replicates, six WT and six *miR-211*−/− RNA samples, according to manufacturer’s instructions (TruSeq RNA Sample Preparation kit), with an initial amount of 4 μg of total RNA.

In particular, we included in the study two males and three females with a miR-211+/+ genotype, and six males with a miR-211−/− genotype. Quality control of library templates was performed using a High Sensitivity DNA Assay kit (Agilent Technologies) on a Bioanalyzer (Agilent Technologies). Qubit quantification platform was used to normalize samples for the library preparation (Qubit 2.0 Fluorometer, Life Technologies). Libraries were sequenced on the Illumina HiSeq. 1000 platform and in 100-nt paired-end format to obtain approximately 30 million read pairs per sample. Reads were aligned and assigned to Gencode^46^ Mouse released M4 transcripts and genes by using RSEM version 1.2.19^45^ with standard parameters. All genes with at least 1 count per million in at least 5 samples were considered for further analysis.

### Data availability

All sequencing data can be found at GEO under the accession code GSE106591. The software code used in this study is available upon request to authors. All other data are available from the authors upon reasonable request.

### Analysis of RNA-Seq datasets

Differentially expressed genes between conditions (*miR-211*−/− vs WT) were detected using the Generalized Linear Model approach implemented in the Bioconductor package “edgeR”^74^. P-value was corrected for multiple hypothesis testing using the FDR procedure. In order to exclude any influence of the sex of the animals on the RNA-seq data, we first compared wt males vs miR-211 KO males. Subsequently we carried out the same analysis on wt females vs. miR-211 KO males. Finally, we extended this comparison to all animals, regardless of their sex. All three analyses yielded comparable results by highlighting the same gene ontology enrichments. The differentially expressed genes (FDR < 0.1) are listed in Supplementary Table 1. Gene Ontology enrichment analysis was performed using the Database for Annotation, Visualization and Integrated Discovery (DAVID) Functional Annotation tool^48^. Gene Set Enrichment Analysis using MSigDb GO gene sets^47^ was performed ranking the genes according to their significance in differential expression. For each GO term the Enrichment Score (ES) and the corresponding p-value were computed by using the KS test function^75^. P-values were corrected for multiple hypothesis testing using the Benjamini-Hochberg procedure. As a positive ES corresponds to GO term containing the best discriminant genes only GO terms with positive ES value and FDR < 5% were considered. Significant GO terms were than ranked according to their p-values.

## Electronic supplementary material

Supplementary Data

Supplementary Table 1

Supplementary Table 2
