# Supplementary material for: MiR-211 is essential for adult cone photoreceptor maintenance and visual function
Source: Sci Rep. 2017 Dec 5;7:17004. doi: 10.1038/s41598-017-17331-z (PMC5717140; doi:10.1038/s41598-017-17331-z)
Supplement: Supplementary file 1 — Supplementary Data [file 41598_2017_17331_MOESM1_ESM.pdf]

MiR-211 is essential for adult cone photoreceptor maintenance and visual function.

Sara Barbato[1], Elena Marrocco[1#], Daniela Intartaglia[1#], Mariateresa Pizzo[1], Sabrina Asteriti[2,§§], Federica Naso[1], Danila Falanga[1], Rajeshwari S. Bhat[1], Nicola Meola[1,§], Annamaria Carissimo[1], Marianthi Karali[1,3], Haydn M. Prosser[4], Lorenzo Cangiano[2], Enrico Maria Surace[1,5], Sandro Banfi[1,3]*, Ivan Conte[1]*

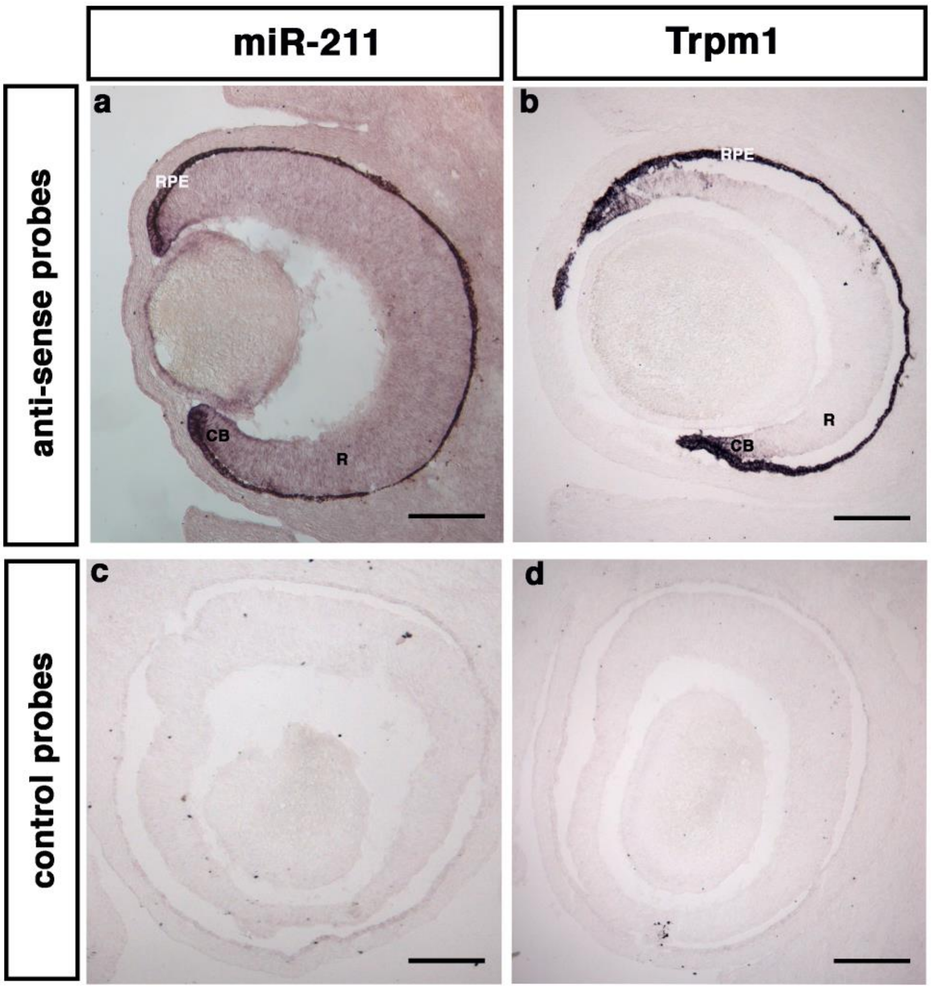

Supplementary Figure 1. MiR-211 and Trpm1 expression.

RNA ISH analysis performed with miR-211 (**a-c**) and Trpm1 (**b-d**) anti-sense probes in E14.5 mouse retina. Scale bars: 10μm. MiR-211 is strongly expressed in the RPE, ciliary body (CB) and the distal region of the retina (R). The *miR-211* host gene, *Trpm1*, displays the same expression pattern. (**c-d)**
RNA ISH experiments performed with control probes for miR-211 (scrambled probe) and for Trpm1 (sense probe).

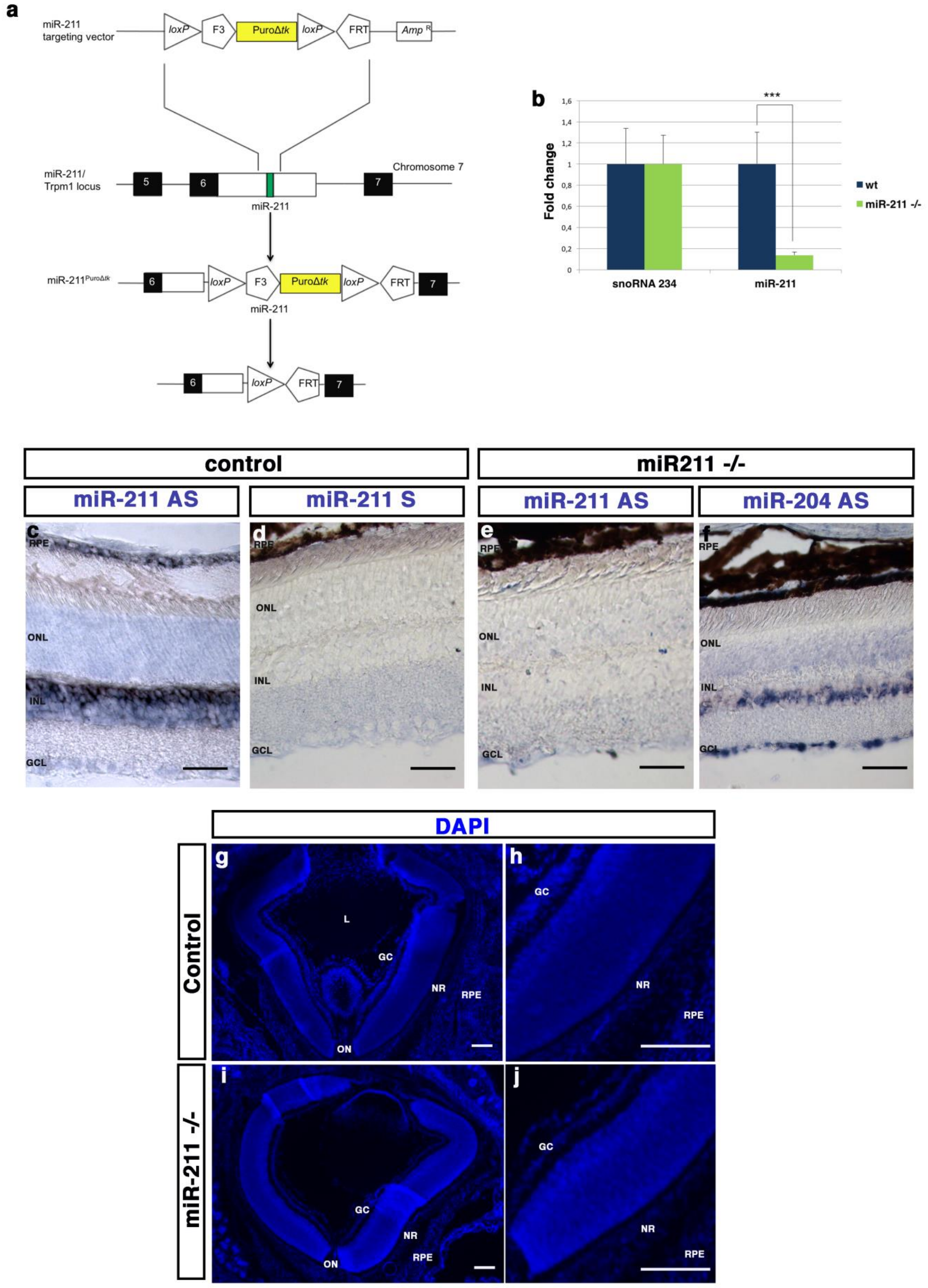

Supplementary Figure 2. Generation of *miR-211*-/- mice and expression of *miR-211*

(**a**) Targeting vector used to generate *miR-211*-/- mouse strain: the *miR-211* gene was removed as a 160 bp deletion using the PuroΔtk cassette. (**b**) qRT-PCR assays for miR-211 show that the mature

miR-211 sequence is not detectable in the retina of *miR-211*-/- mice. Error bars represent SEM. ***P Value < $3x10^{-6}$ t test. (**c-f**) Expression profile of *miR-211* in wt mouse retina: (**c**) RNA ISH with a miR-211 probe on sections from mouse adult retina. Expression signal is detected in the ganglion cell layer (GCL), in the inner nuclear layer (INL), in the outer nuclear layer (ONL) and in the retinal pigment epithelium (RPE). (**d**) RNA ISH with a scrambled (S) miRNA probe (negative control). (**e**) RNA ISH with a miR-211 probe on sections from *miR-211*-/- retina does not reveal the presence of any non-specific signal that could be ascribed to the paralogous miR-204 sequence. **(f)** RNA ISH with a miR-204 probe on sections from *miR-211*-/- retina. (**g-j)** Representative immunofluorescence images of P1 control (**g, h)** and *miR-211*-/- (**i, j**) retinas, stained with DAPI (blue). L (lens); GC (ganglion cells); NR (neural retina); ON (optic nerve); RPE (Retinal Pigment Epithelium). Scale bars: 50μm.

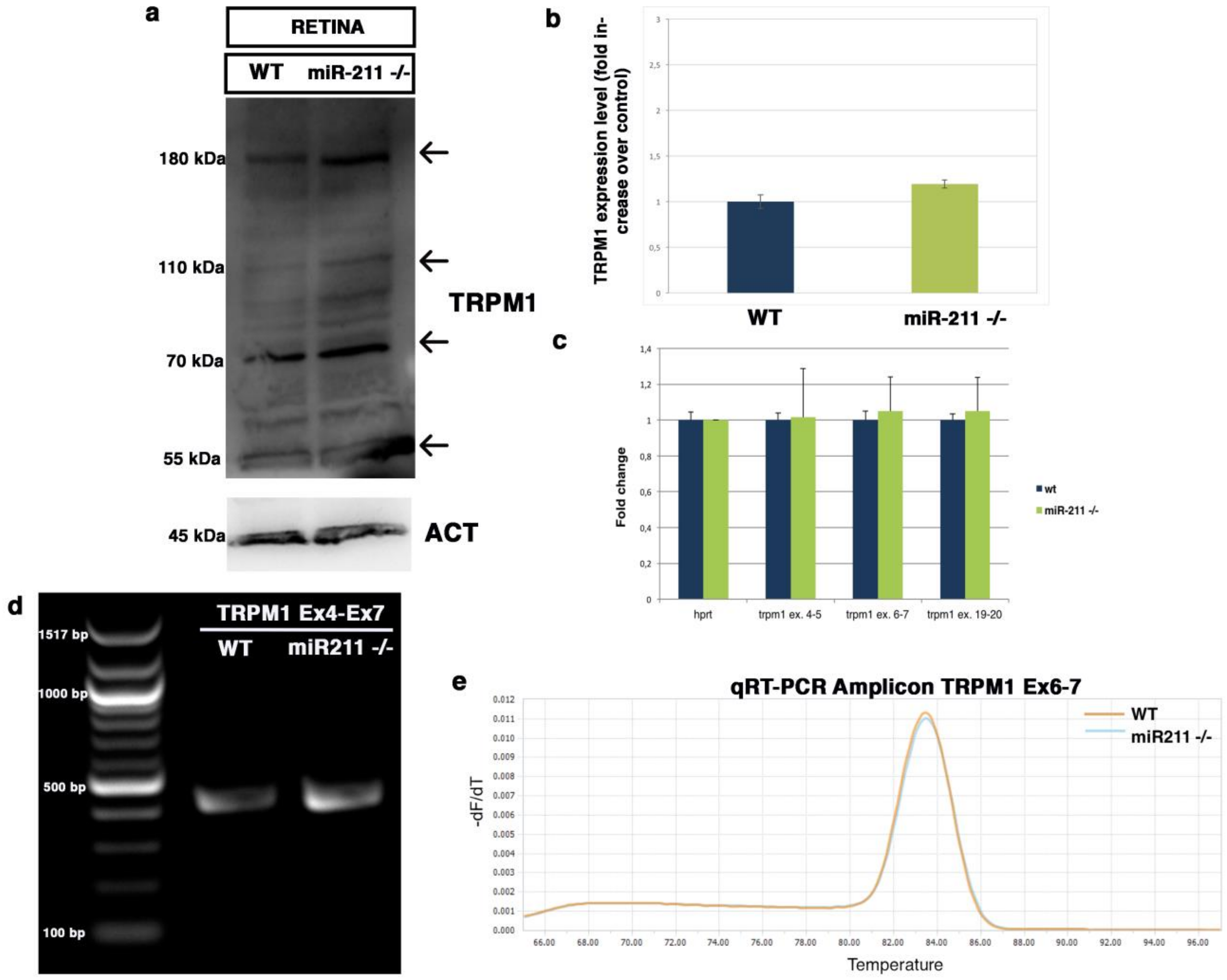

Supplementary Figure 3. No impact of miR-211 deletion on the expression of the Trpm1 host gene

(**a, b**) Representative Western blot analysis on retina from wt and *miR-211*-/- animals [the arrows show representative TRPM1 bands (i.e. 180 kDa, 110 kDa, 70 kDa and 55 kDa) previously reported in (29)]. (**b**) Quantification of the data reported in (**a**). (**c**) qRT-PCR analysis on total RNA extracted from whole eye samples, after lens and cornea removal, shows that TRPM1 expression levels are not altered in the *miR-211*-/- mice when compared to wt controls. Error bars represent SEM. (**d**) RT-PCR analysis does not indicate any alteration in the maturation of *Trpm1* mRNA. **(e)** The profiles of Exon 6-7 amplicon products in qRT-PCR analysis are fully overlapping between *miR-211*-/- and wt eyes, further supporting a correct splicing activity of the miR-211-containing intron of *Trpm1*.

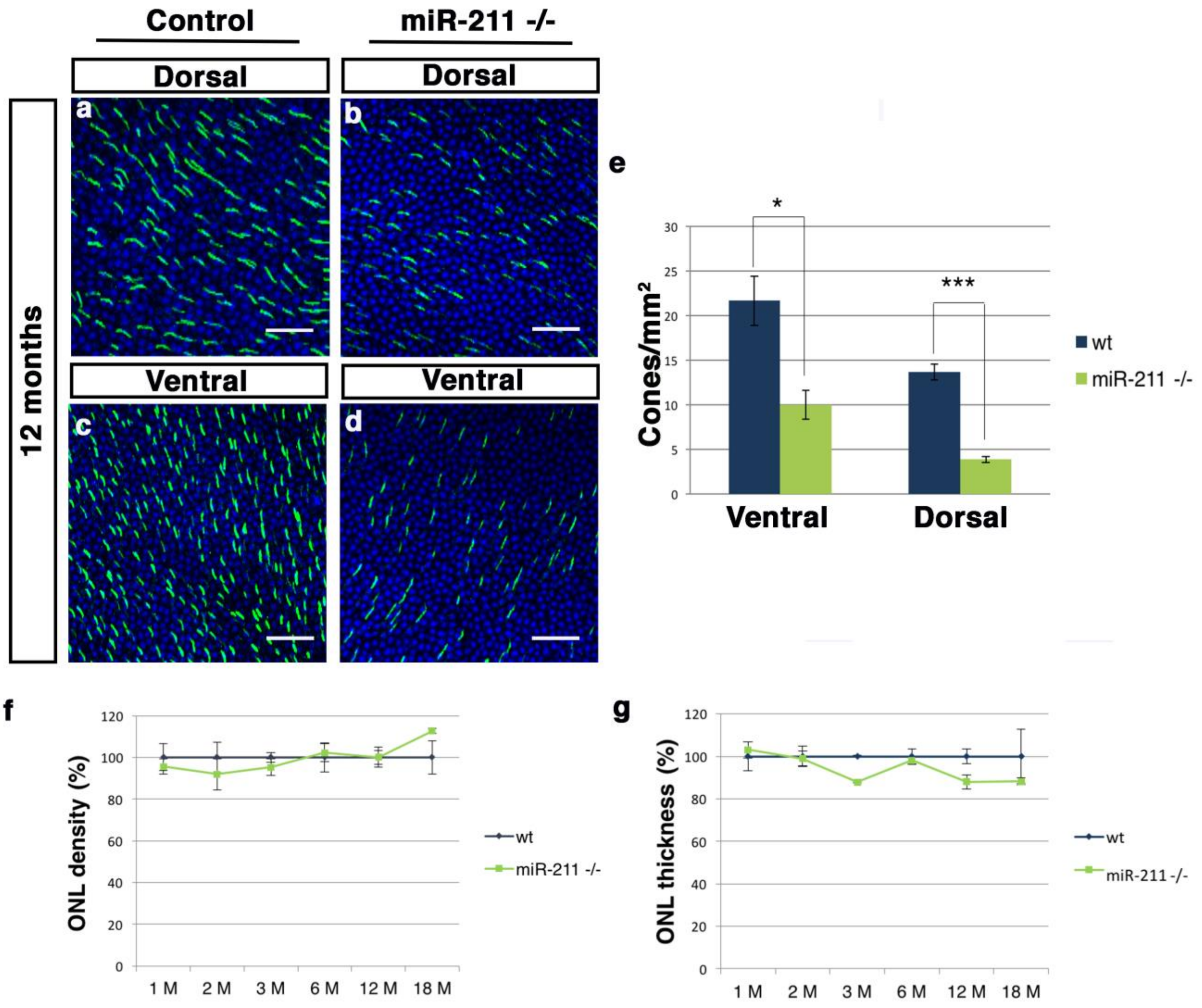

Supplementary Figure 4. Cone dysfunction undergoes progressive worsening over time in *miR-211*-/- mice.

(a-d) Representative retina flat mount images from wt (**a-c**) and *miR-211*-/- (**b-d**) at twelve months, stained with cone-Arrestin (green) and DAPI (blue). Scale bars: 25µm. Cone-Arrestin signal is significantly decreased in *miR-211*-/- compared to controls at each of the selected time points. **(e)** Graph showing cone density (cones/area) in wt and *miR-211*-/- mice at twelve months. Error bars represent SEM. *P Value < 0.05, ***P Value < 0.001. (**f-g**). No significant differences are visible in the number of rods and in ONL density and thickness between miR-211-/- and wt mice. Error bars represent SEM. ONL (Outer Nuclear Layer).

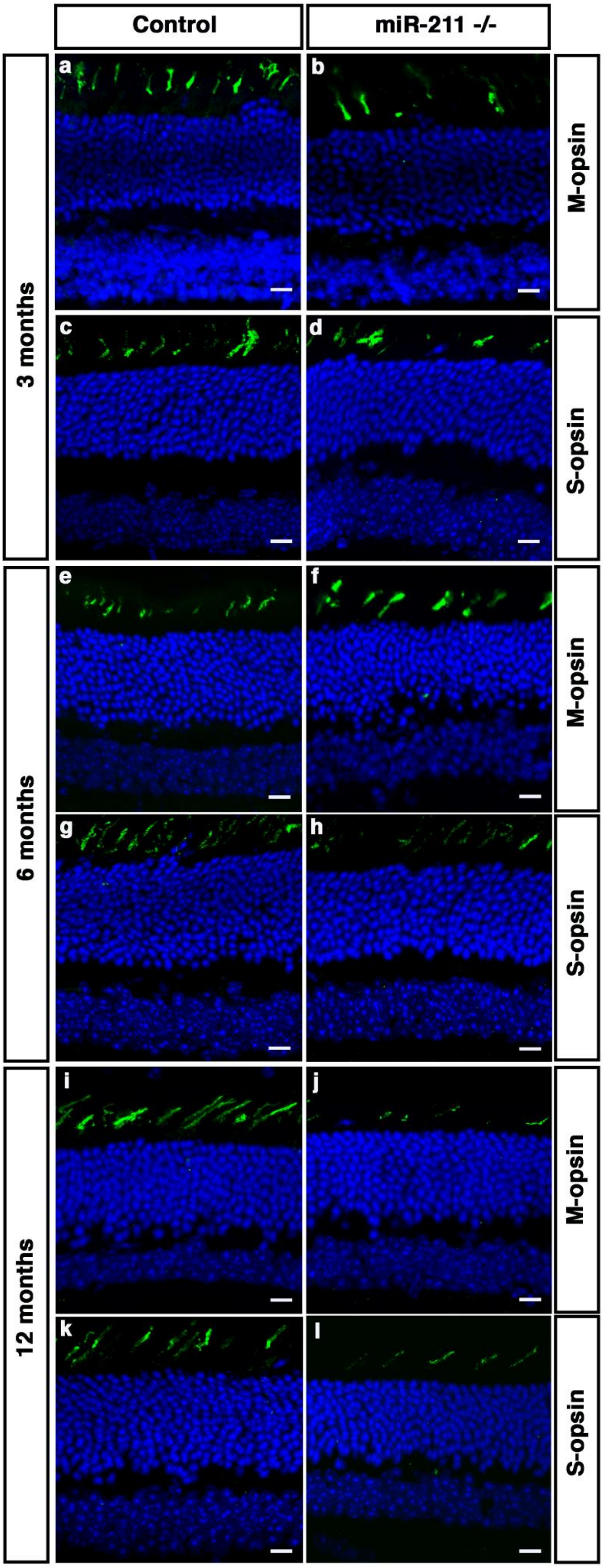
Control
miR-211 -/-
3 months
6 months
12 months
M-opsin
S-opsin
M-opsin
S-opsin
M-opsin
S-opsin
a
b
c
d
e
f
g
h
i
j
k
l

Supplementary Figure 5. Cone opsin expression decrease over time in *miR-211*-/- mice.

(a-l) Representative immunofluorescence images of control **(a, c, e, g, i, k)** and *miR-211*-/- **(b, d, f, h, j, l)** retinas at three months **(a-d)**, six months **(e-h)** and twelve months **(i-l)** of age stained with anti-M- and anti-S-opsin antibodies (green) and DAPI (blue). The signal of both M-opsin and S-opsin shows a progressive decrease of signal over the time in *miR-211*-/- mice as compared to controls. Scale bars: 50μm.

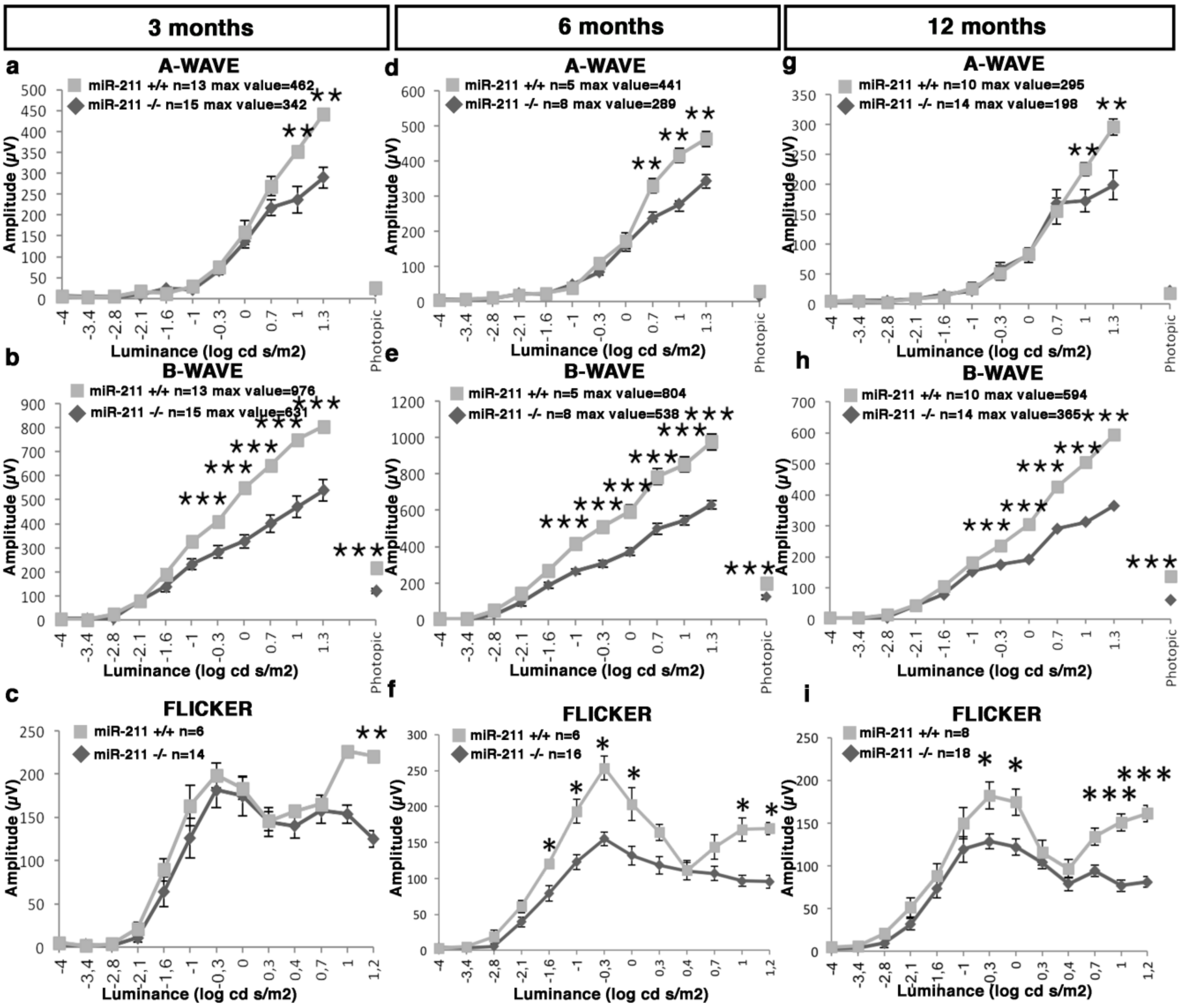

Supplementary Figure 6. miR-211 deletion induces progressive reduction in cone photoreceptor response.

(**a, b, d, e, g, h**) ERG responses, plotted as a function of stimulus intensity, from wt (grey squares) and *miR-211*-/- (black rhombi) mice, at three months **(a-b)**, six months **(d-e)** and one year **(g-h)** of age. The amplitude of the dark-adapted, scotopic a-wave and b-wave of the *miR-211*-/- mice are significantly lower than in wt control mice. Error bars represent SEM. **(c, f, i)** Flicker responses, plotted as a function of stimulus intensity, from wt (grey squares) and *miR-211*-/- (black rhombi) mice, at three **(c)**, six months **(f)** and one year **(i)** of age. The b-wave amplitude of *miR-211*-/- mice is significantly lower than wild type mice, in the light intensities range that stimulates mainly cone response. At six months of age *miR-211*-/- mice show a decrease of b-wave amplitude in the light intensities range that stimulates not only cone but also some rod response. Photopic indicates fleshes of 20.0 cd s/m^2 light intensity on a constant background illumination of 50 cd/m^2. Error bars represent SEM. *P Value < 0.05, **P Value < 0.01 t test, ***P Value < 0.001.

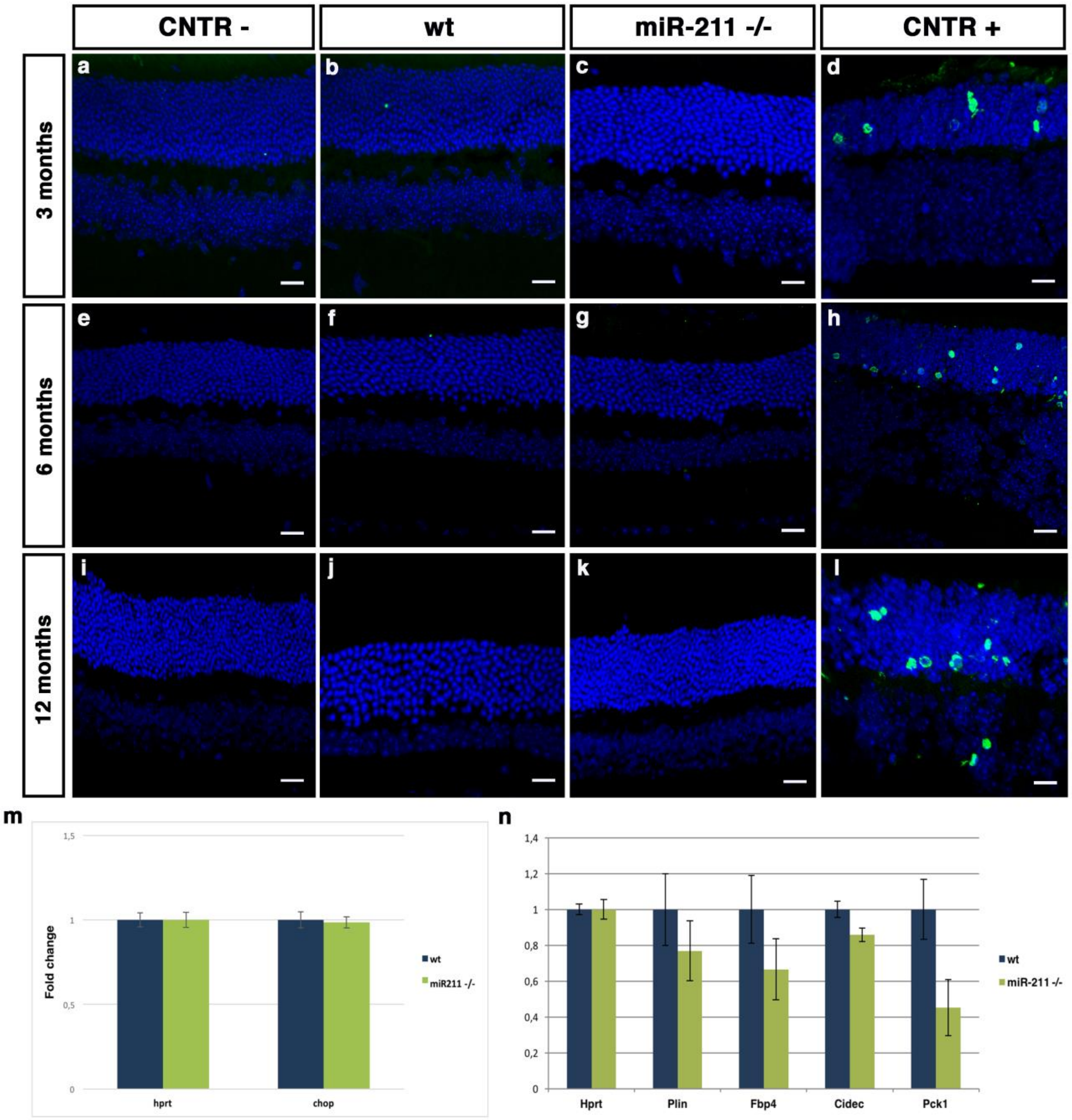

Supplementary Figure 7. Evaluation of cell death in the retina of *miR-211*-/- mice.

(**a-l**) Representative images of (**a, e, i**) negative control (CNTR-), (**b, f, j**) wt, (**c, g, k**) miR-211-/- and (**d, h, l**) positive control (CNTR +) retinas, at three months (**a, b, c, d**), six months (**e, f, g, h**) and twelve months (**i, j, k, l**), stained with TUNEL-fluorescein (green) and DAPI (blue). No difference in the number of apoptotic cells is detected between wt and *miR-211*-/- mice at six months of age. Note that CNTR – are retina sections incubated with the reaction mix without TUNEL reaction enzyme and CNTR + are retina sections from *Apl1*-/- mice at P12. The *Apl1*-/- mice is characterized by a high extent of photoreceptor cell death[65,66]. Scale bars: 100μm. **(m)** qRT-PCR analysis on cDNAs from mouse eye samples, after removal of lens and cornea, shows no differences in Chop expression between wt and *miR-211*-/- mice at 1 month of age. **(n)** qRT-PCR analysis on cDNAs from mouse retina samples shows the differences in the expression of *Plin*, *Fbp4*, *Cidec* and *Pck1* genes in line with our RNAseq data (see Supplementary Table 1). Error bars represent SEM.

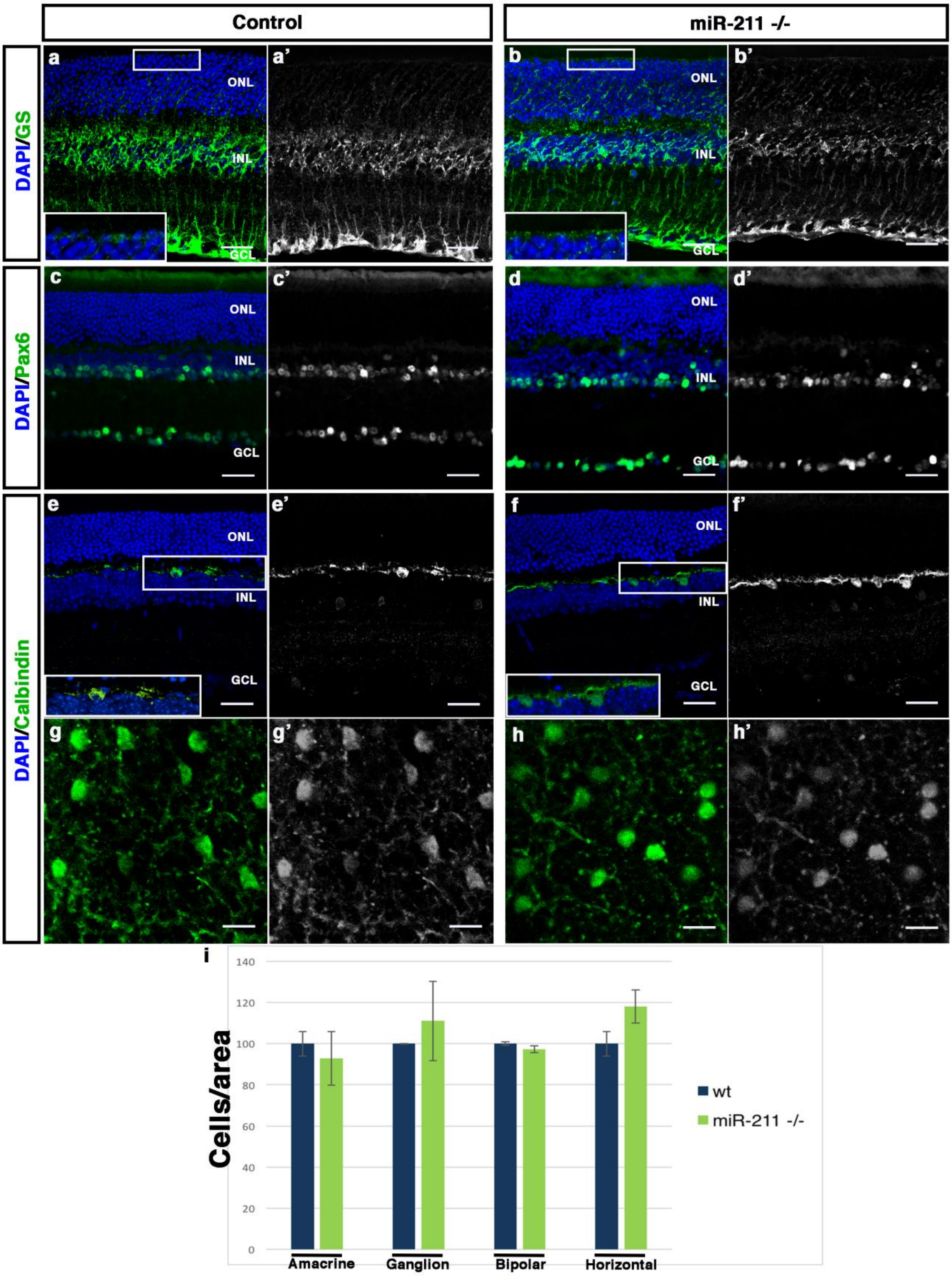

Supplementary Figure 8. MiR-211 ablation does not lead to alteration of other retina cell types.

Adult (7 months old) mouse retina sections from wt **(a, a', c, c', e, e', g, g')** and *miR-211*-/- **(b, b', d, d', f, f', h, h')** mice were immunostained with antibodies against the Glutamine Synthase (GS) **(a, a', b, b')**, Paired Box 6 (Pax 6) **(c, c', d, d')** and Calbindin **(e, e', f, f', g, g', h, h')** proteins, which are marker for Müller glial, ganglion and amacrine cells and horizontal cells, respectively. (**g,**

g', h, h') Representative retina flat mount images from wt (**g-g'**) and *miR-211*-/- (**h-h'**) at six months of age, stained with Calbindin (green) and DAPI (blue). ONL (Outer Nuclear Layer), INL (Inner Nuclear Layer), GCL (Ganglion Cell Layer). Scale bars: 50µm. (**i**) Graphs showing no differences in the number of amacrine cells, ganglion cells, bipolar cells and horizontal cells between *miR-211*-/- and wt mice. Error bars represent SEM.

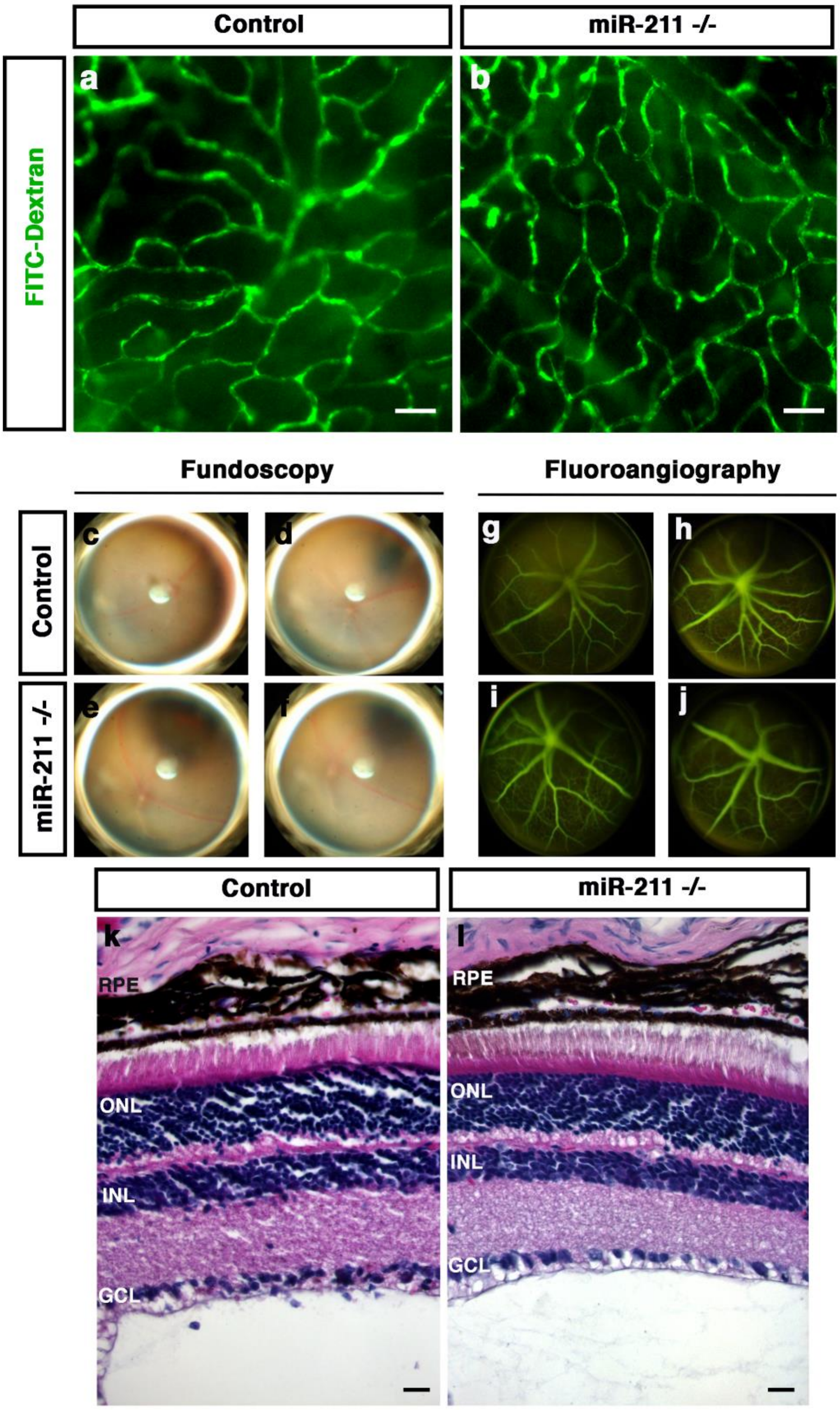

Supplementary Figure 9. Retinal circulation and vasculature are not impaired in *miR-211*-/- mice.

(**a-b**) Representative retinal flat mount images from six months-old wild type (**a**) and *miR-211*-/- (**b**) mice, after perfusion with fluorescein isothiocyanate (FITC)-dextran. *miR-211*-/- inner retinal vasculature do not show edema end/or neovascularized areas, and display similar shape and distribution pattern with respect to control mice. Scale bars: 1μm. (**c-j**) Representative in vivo images of ocular fundus in one year-old mice. The analysis, carried out by fundoscopy (**c, d, e, f**)

and by fluoroangiography (**g, h, i, j**) reveals no abnormalities in retinal vessels and pigmentation in *miR-211*-/- (**e, f, i, j**) with respect to wt mice (**c, d, g, h**). **(k-l)** Representative images of ematoxylin and eosin-stained retina sections of wt **(k)** and *miR-211*-/- **(l)** mice. (RPE) Retinal Pigment Epithelium; (ONL) outer nuclear layer; (INL) inner nuclear layer; (GCL) ganglion cell layer. Scale bars: 50μm.
